# Release of Bisphenol A and Other Volatile Chemicals from New Epoxy Drinking Water Pipe Liners: The Role of Manufacturing Conditions

**DOI:** 10.1021/acs.est.4c08663

**Published:** 2025-01-02

**Authors:** Pritee Pahari, Samuel Spears, Jianghui Liu, Sydney Butler, Shantanu Sonowane, Anthony Garcia, Madeline Larsen, Caitlin R. Proctor, John A. Howarter, Jeffery Paul Youngblood, Nusrat Jung, Andrew J. Whelton

**Affiliations:** 1 Lyles School of Civil Engineering, Purdue University, 550 Stadium Mall Drive, West Lafayette, Indiana 47907, United States; 2 Division of Environmental and Ecological Engineering, Purdue University, 610 Purdue Mall, West Lafayette, Indiana 47907, United States; 3 Lyles School of Civil Engineering, Purdue University, 550 Stadium Mall Drive, West Lafayette, Indiana 47907, United States; 4 Department of Civil and Environmental Engineering, Howard University, 2300 Sixth Street NW, Washington, D.C. 20059, United States; 5 Division of Environmental and Ecological Engineering, Purdue University, 610 Purdue Mall, West Lafayette, Indiana 47907, United States; 6 School of Materials Engineering, Division of Environmental and Ecological Engineering, Purdue University, 610 Purdue Mall, West Lafayette, Indiana 47907, United States; 7 Division of Environmental and Ecological Engineering, Purdue University, 610 Purdue Mall, West Lafayette, Indiana 47907, United States; 8 Division of Environmental and Ecological Engineering, Purdue University, 610 Purdue Mall, West Lafayette, Indiana 47907, United States; 9 School of Agricultural and Biological Engineering, Division of Environmental and Ecological Engineering, Purdue University, 610 Purdue Mall, West Lafayette, Indiana 47907, United States; 10 School of Materials Engineering, Purdue University, 701 West Stadium Avenue, West Lafayette, Indiana 47907, United States; 11 Lyles School of Civil Engineering, Purdue University, 550 Stadium Mall Drive, West Lafayette, Indiana 47907, United States; 12 Lyles School of Civil Engineering, Division of Environmental and Ecological Engineering, Purdue University, 550 Stadium Mall Drive, West Lafayette, Indiana 47907, United States

**Keywords:** plastic, drinking water, leaching, bisphenol, rehabilitation

## Abstract

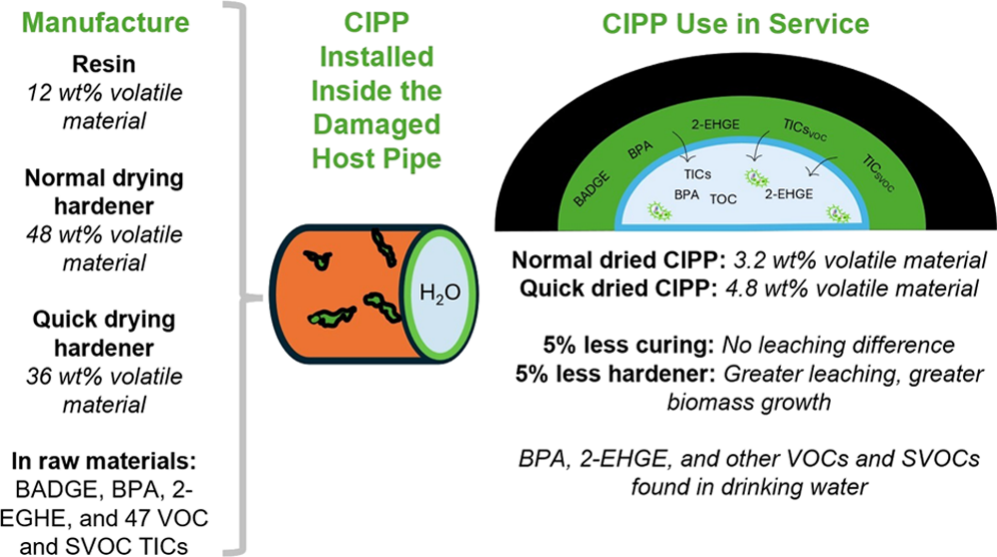

Cured-in-place-pipe (CIPP) technology has begun to be adopted for drinking water pipe repairs, and limited information exists about its drinking water quality impacts. CIPP involves the manufacture of a new plastic pipe inside a buried damaged pipe. In this study, the chemical composition of the raw materials and CIPP water quality impacts were examined. Numerous (47) VOCs and SVOCs were found in the resin and two hardeners were studied. TGA results indicated that new CIPPs contained about 3.2–4.8 wt% VOC. A controlled static headspace analysis using PTR-TOF-MS detected eight protonated ions (*m*/*z* < 114) with mixing ratios above 1 ppb and phenol was identified as the most abundant VOC released into air. The analytical methods were unable to identify 99.9% of the extractable VOC mass. A 5% shorter curing duration had no impact on the chemical residual left in the CIPPs, but a 5% hardener reduction prompted an increase in the amount of monomer remaining. Both normal drying and quick drying formulations released similar amounts of 2-EHGE and BPA into drinking water. Laboratory results scaled to 4–36 in. diameter pipes indicated only 4 in. diameter CIPPs would cause BPA levels to exceed a U.S. state drinking water standard, EU, and WHO drinking water limits. Flushing and drinking water VOC and SVOC testing should be conducted before use. Additional studies of resins, manufacturing conditions, and wastes generated, are recommended. At present, the limited information available about these materials has shifted the burden of potential health and financial costs to the users.

## Abstract 2

Limited information exists regarding the drinking water quality impacts of cured-in-place-pipe (CIPP) technology. Water quality impacts were found.

## 1 Introduction

To lessen the cost of aging drinking water pipe repairs in the U.S. and Europe, some utilities have begun using cured-in-place-pipe (CIPP) technology.^1−3^ CIPP technology involves the insertion of a resin-saturated tube into an existing damaged pipe. The resin is then transformed into a hard plastic by exposure to hot water, steam, ultraviolet radiation, or ambient air.^4^ Epoxy is the most popular resin for drinking water CIPP.^4^ Some, but not all, CIPPs have inner plastic films (i.e., polypropylene, polyethylene, and polyurethane) separating the resin from drinking water. This technology has been proposed for rehabilitating 4 to 96 in. diameter water mains^5^ and even lead service lines.^6^

Limited water quality information exists about drinking water CIPPs globally, and chemicals used for CIPP manufacture have not been widely reported. A review of some U.S. materials revealed that many ingredients lack drinking water standards (Table 1). It is unclear what degree CIPP ingredients or chemicals produced during manufacture leach into drinking water. For example, bisphenol A diglycidyl ether (BADGE) is a popular epoxy monomer and does not have a U.S.-regulated drinking water standard. The leaching of bisphenol A (BPA), a reactant for BADGE synthesis, has been a concern for drinking water in Europe. In 2016, Sweden banned BPA use for drinking water pipes^7^ and the European Union subsequently set a 2.5 μg/L BPA drinking water limit.^8^ Like BADGE, BPA does not have a U.S. drinking water limit, but the state of Minnesota imposed short- (100 μg/L) and long-term (20 μg/L) BPA drinking water guidance values.^9^ To reduce the chance that drinking water contact materials pose a health risk in the U.S., product certification with ANSI/NSFI standard 61 has been encouraged.^10^ For certification, the product is subjected to three sequential 24 h water exposure periods at 23 °C, and then, water from the third period is chemically analyzed. Product manufacturing, handling conditions, and certification drinking water test results are not made public. This is a major challenge in understanding why drinking water contamination is caused. Some CIPP manufacturers have claimed certification demonstrates that their products “do not leach contaminants into the water that would be a health concern”.^11^ Another is that certification “ensure[s] water meets all standards set by the ··· *Safe Drinking Water Act*.”^12^ Others claim the liner will not leach any chemicals into drinking water.^13^

**Table 1:** Few Chemicals Reported on Material Safety Data Sheets for the Raw Materials Used for Drinking Water CIPP Manufacturing in the U.S. Have Been Evaluated for Federal Drinking Water Standards^a^

		**products reported on SDS documents, % unless shown otherwise**									
**listed ingredient**	**CASRN**	**A**	**B**	**C**	**D**	**E**	**F**	**G**	**H**	**I**	**J**
polyamides	63428–83–1				>80						
bisphenol A reaction product	25085–99–8			<85							
epoxy resin	25068–38–6						50–80	40–70	50–80	10–30	10–30
4,4’-isopropylidenediphenol-epichlorohydrin copolymers	25068–38–6	55–90									
fatty acids, C18-unsatd., dimers, reaction products with polyethylenepolyamines	68410–23–1		30–65								
teta, reaction products with phenol and formaldehyde	32610–77–8					40–70					
triethylenetetramine	112–24–3		30–50			15–40					
*phenol	108–95–2					15–40					
*benzyl alcohol	100–51–6				<15						
polyglycol diglycidyl ether modifier	74398–71–3							5–15			
polyfunctional glycidyl ether modifier	26142–30–3									5–15	5–10
polyglycol diglycidyl ether	26142–30–3						5–15		5–15		
2-ethyl hexyl glycidyl ether (EHGE)	2461–15–6			>10							
*xylene	1330–20–7									<7	7–13
isophoronediamine	2855–13–2				>5						
epichlorohydrin	106–89–8	3–4 ppm									
carbon black	1333–86–4						<2		<2		
*acetone	67–64–1									<1	
petroleum distillates	64742–47–8			<1							
adhesion promoter	not reported									<1	

a Letters A–J indicate separate products (resins and hardeners) marketed for drinking water epoxy CIPP. The asterix (*) symbol indicates a U.S. federal drinking water maximum contaminant level or health advisory level exists.

Only a few CIPP drinking water testing studies were found in the literature, and all lacked the information needed for broad conclusions. The most noteworthy study was from California, where four ANSI/NSF 61 certified epoxy CIPPs caused drinking water to become milky white and foam (Figure S1). These phenomena prompted the public utility to keep the CIPPs out of service and conduct weeks of water flushing.^14,15^ Due to water toxicity concerns, the wastewater utility prohibited flush water discharge to their sewer. This drove the drinking water utility to collect and treat the flush water with activated carbon and then haul it to a wastewater treatment plant. Flush water contained acetone, methyl ethyl ketone, 4-methyl-2-pentanone, styrene, and phthalates. Waste disposal costs were approximately $250,000 instead of the $15,000 estimated project cost before the flush water toxicity concerns.^16^ Other drinking water sampling efforts for 6 to 16 in. diameter CIPPs in Ohio and Wisconsin detected BPA (0.02 μg/L to 20.8 μg/L), di(2-ethylhexyl) phthalate (1.39 to 3.03 μg/L), di(2-ethylhexyl) adipate (0.7 to 1.27 μg/L), and methylene chloride (1.06 μg/L).^17−19^ Most recently, in 2018, a consultant reported epoxy CIPP ANSI/NSFI Standard 61 chemical leaching results as “<40 μg/L BADGE, 1 μg/L trichloromethane, <5 μg/L dibutylphthalate, <5 μg/L dodecanol, <3 μg/L toluene, <1 μg/L *m-, p-*xylenes, and >300 μg/L 2-ethylhexyl glycidyl ether (EHGE)”.^2^ In Italy, testing revealed that BPA reacted with chlorine disinfectant and epoxy CIPP deterioration prompted increased BPA leaching.^20^

Unlike the paucity of data for epoxy CIPP, many studies have documented drinking water quality impacts of epoxy tank and pipe coatings, even for ANSI/NSFI Standard 61 certified materials. Total organic carbon (TOC) levels have been found as high as 345 mg/L^22^ for a certified product, and in one study, water prerinsing had little effect on leaching.^22^ Chemicals such as BPA, BADGE, alcohol, phenolic, phthalate compounds, and others have been observed to leach from epoxy coatings^20,21,23−35^ (Tables SI-1–3). Leached coating compounds react with free chlorine disinfectant^18,30,34,35^ forming disinfectant byproducts,^36^ causing drinking water tastes,^38^ “plastic, glue, putty” odors for 30 days,^36^ and increased microbial growth.^26^ It remains unclear the extent of chemical leaching similarities shared between epoxy coatings and epoxy CIPP liners. Evidence suggests that CIPPs could impact drinking water quality, and this necessitates a need for understanding.

To better understand CIPP drinking water quality impacts, the chemical composition of raw materials and CIPP composites was examined. Composites were created under standard and nonstandard conditions to explore the impact of manufacturing conditions on water quality impacts. Specific objectives were to (1) identify volatile organic compounds (VOC) and semivolatile organic compounds (SVOC) in the resin and hardeners, (2) characterize chemical residuals in CIPP composites, (3) examine the effect of nonstandard manufacturing conditions on composite chemical residual, and (4) quantify the chemicals leached into drinking water. For the first time, proton transfer reaction time-of-flight-mass spectrometry (PTR-TOF-MS) was applied to speciate the VOCs emitted into air from new CIPP composites. This measurement technique has been applied to detect VOCs emitted from indoor and outdoor industrial sources.^39−47^

## 2 Materials and Methods

### 2.1 Materials

Polymer composites were manufactured by using Rhino Lining Corporation resin (1310T-LT BLUE-G) and two hardeners (3102 and 3191 epoxy hardener). According to the material Safety Data Sheet, the resin contained 75 to 80% BPA epichlorohydrin epoxy resin, 10 to 20% [[(2-ethylhexyl) oxy] methyl] oxirane, and 1 to 5% silicon dioxide; 3102 normal drying hardener (referred to here as the NDry Hardener) contained more than 80% polyamides, less than 5% benzyl alcohol, and more than 5% isophoronediamine; 3191 quick drying hardener (referred to here as the QDry Hardener) contained 55 to 65% reaction products with phenol and formaldehyde, 15 to 20% triethylenetetramine, and phenol (Table SI-4).^48^ Polyester felt (model 24–125 PE, Sutherland Felt Company, USA) of 0.2 cm thickness was the reinforcement. A perforated Teflon film (ACP Composites, Inc., USA) created a barrier between the stainless steel and felts during the curing process.

Analytical standards and solvents were used for chemical identification, quantification, and liquid–liquid and solid–liquid extractions. Individual 2-EHGE, BPA, and BADGE standards were procured from Sigma-Aldrich. Methanol, dichloromethane, acetone, sodium chloride, chlorobenzene-*d*_5_, BPA-*d*_16_, and naphthalene-*d*_8_ were sourced from Thermo Fisher Scientific, Inc. A mixed standard consisting of 100 mg/L dissolved in acetone was prepared. Laboratory-prepared water with a resistivity of 18.2 MΩ cm (Thermo Scientific Barnstead nanopure water purification system) was utilized for experiments.

### 2.2 Composites Were Manufactured Using Dry Heat

To determine whether slight changes in manufacturing conditions influence chemical release from new CIPP composites, the authors adopted one standard and two nonstandard manufacturing conditions. The resin-to-hardener ratios of the standard manufacturing conditions for the NDry Hardener and QDry Hardener were 4:1 and 2:1, respectively. Composites that were manufactured using the NDry Hardener and the QDry Hardener are referred to as the NDry Composite and QDry Composite, respectively. To explore the sensitivity of chemical leaching due to nonstandard manufacturing conditions, one nonstandard composite was manufactured using a 5% shorter curing time. Another nonstandard composite was created using 5% less hardener mass compared to the amount recommended by the manufacturer. Some variability in resin and hardener ratios is permitted on ANSI/NSFI 61 certified products^49^ (i.e., ± 2%), but a greater difference was adopted here so that some products would be out of compliance. The resin and hardener mixtures that were prepared are described in the SI.

### 2.3 Chemical Analysis of Resin, Hardeners, and Composites

The volatile content (wt%) of resin, hardeners, and composites was measured using a Q-500 instrument from TA Instruments, Inc. (Delaware, USA). Each 20 to 40 mg thermogravimetric analysis (TGA) sample was subjected to heating in a platinum pan at a rate of 10 °C per min until reaching 160 °C. The temperature was maintained for 120 min in a controlled nitrogen atmosphere with a purge flow rate set at 60 mL/min. This approach was like that applied by others who have estimated VOC content in sewer CIPPs.^50−52^

Volatile compound release from the surface of the composite into air was separately measured using a ppbRAE photoionization detector (PID) (10.6 eV lamp, RAE System, USA) and PTR-TOF-MS (PTR-TOF 6000, Ionicon Analytik Ges.m.b.H., Austria). The experimental setup for the PID sampling and PTR-TOF-MS headspace experimental sequence can be found in the Supporting Information (SI) file. For the PTR-TOF-MS headspace sampling approach, 0.002 g of QDry composite pieces were prepared in a 20 mL sampling vial. QDry composites were the focus of the PTR-TOF-MS examination because they contained a greater mass of volatile material than the NDry composites.

### 2.4 Solid–Liquid Extraction of Resin, Hardeners, and Composites

Resin, hardeners, and composites underwent solid–liquid extraction, and extracts were analyzed by gas chromatography–mass spectrometry (GC-MS). Extractions were conducted by dissolving 3 g of one material into DCM in a 20 mL amber vial at 23 °C for 72 h. Solid–liquid extraction has been used to chemically characterize CIPP composites previously.^50,69^ Composite extractions were carried out using material drilled from plates to enhance the surface area. After the 72 h extraction period, aliquots were removed, diluted to 1:100 and 1:10, and analyzed using the GC-MS.

### 2.5 Chemical Leaching into Drinking Water

Composites were immersed in laboratory-prepared Eastern U.S. soft drinking water pH 7–8 (SI) for a total of 168 h, with periodic water changes. In 600 mL jars, two 5.08 × 5.08 × 2.25 cm composites (in wedge formation to increase surface area) were placed in 525 mL of drinking water headspace-free. The total surface area was 116 cm^2^ resulting in a surface area to volume ratio of 0.22 cm^2^/mL. Leaching was carried out for three sequential 24 h periods, followed by a final leaching period of 96 h. The final period was conducted to increase the chance of finding contaminants leaching from the composite if the shorter 24 h periods did not reveal detectable compounds. After each leaching period, TOC concentration and chemical identification and quantification measurements were conducted. Extractions were conducted like that described in USEPA Method 3511.^53^ A 5 mL aliquot from a 40 mL amber vial was mixed with naphthalene-*d*_8_ (918 mg/L) and BPA-*d*_16_ as surrogate standards. DCM (2 mL) and sodium chloride (6 g) were added, the vial was sealed and agitated, and 1 mL of DCM was withdrawn into a 1,500 μL GC vial for analysis. Chlorobenzene-*d*_5_ served as the internal standard for quantification. A test to analyze the potential for leachate to support biological growth was also performed, modeled after the standard BioMig biomass formation potential test for materials testing.^54^ A description of the methods for this analysis can be found in the SI section.

Composites did not undergo disinfection prior to leaching measurements for several reasons. First, the manufacturer stated that the pipes were ready for service following the curing period. Also, because the shock disinfection method applied can alter the chemical leaching of other plastic pipes,^55^ that variable was not introduced here. Finally, free chlorine reacts with BPA, so chlorination may transform and degrade BPA on the CIPP surface, thereby inhibiting the detection of BPA release from the new products.

### 2.6 Chemical Characterization

Chemical assessment of uncured resin, hardeners, and composites was conducted using a Shimadzu 2010-Plus GC-MS (Shimadzu, Inc., USA) employing an HP-5MSUI capillary column with dimensions of 30 m in length, 0.25 mm in diameter, and a 0.25 μm film thickness. Helium was employed as the carrier gas flowing at a rate of 1.5 mL/min. The temperature program for the GC oven was as follows: an initial temperature of 40 °C (maintained for 4 min), followed by a ramp to 300 °C at a rate of 9.6 °C/min (maintained for 5 min), using the direct injector mode. The injection was split less, and high pressure and the injector temperature was maintained at 300 °C. The purge flow and column flow rates were set at 5 mL/min and 1.20 mL/min, respectively. The total program run time was 36 min. In MS, the ion source temperature was 250 °C and the interface temperature was 290 °C. The initial scan method assessed tentatively identifying those matching over 70% in the NIST database. Subsequently, the selected ion monitoring method targeted three compounds (2-EHGE, retention time 15.1 min; BPA, retention time 25.5 min; BADGE, retention time 31.6 min). 1,4-Dichlorobenzene-*d*_4_ (1 mg/L) was used as an internal standard. Calibration curves for 2-EHGE, BPA, and BADGE were constructed from 0.1 to 10 ppm utilizing standards (98, 99, and >95% purity, respectively) and dichloromethane.

TOC concentration was determined by applying the USEPA Method 415.3^56^ and a TOC-LCPH TOC analyzer (Shimadzu, Shanghai). Nitric acid-washed carbon-free vials were utilized for sample collection. A calibration curve was established using potassium phthalate (0.1 to 5 mg/L: *r*^2^ = 0.99). Samples stored at 4 °C were analyzed within seven days of collection. Prior to storage, 200 μL of 3 N hydrochloric acid was added to 20 mL of each sample.

### 2.7 Statistical Methods

A one-way analysis of variance (ANOVA) was employed to assess the significance of differences among the sample means. Student *t* tests were conducted for pairwise comparisons in cases involving only two groups. Pairwise comparisons were made with the Tukey studentized range for data sets exhibiting significant ANOVA results. Significance was established at *p* < 0.05.

## 3 Results and Discussion

### 3.1 Epoxy Resin and Hardeners Contained Notable Quantities of VOCs and SVOCs

TGA and GC-MS results revealed that all materials contained notable quantities of VOCs and SVOCs. The hardeners contained a significantly greater amount of volatile material [NDry Hardener (48.13 wt%) and QDry Hardener (36.43 wt%)] than the resin (12.72 wt%) (*p* = 0.009) (Figure 2). The high standard deviation values detected for the hardeners (±15 to 27 wt%) indicate heterogeneity, in comparison to homogeneity observed for the resin (±0.20 wt%). NDry and QDry Composites had a much lower amount of volatile material (3.2 wt%, 4.8 wt%) than the hardeners and resin (Table S2).

**Figure 1:**
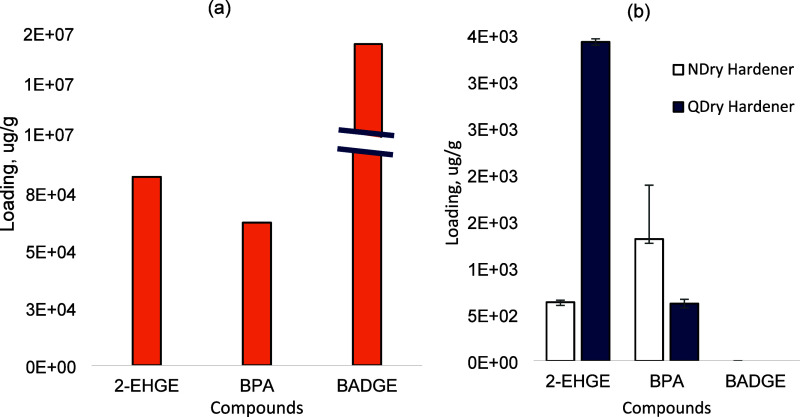
The (a) resin contained higher loadings of monomer and viscosity reducer compared to the (b) hardeners. BADGE was only detected in the resin, but BPA and 2-EHGE were detected in both the resin and hardeners. Mean and standard deviation values are shown for three replicates.

Both the resin and hardeners contained 2-EHGE, BPA, and BADGE was present only in the resin (Figure 1). 2-EHGE is a viscosity reducer and a VOC with a boiling point of 120 °C.^57,58^ 2-EHGE was previously reported to leach from an ANSI/NSFI standard 61 certified epoxy CIPP.^2^ Interestingly, the QDry Hardener contained three times more 2-EHGE mass than the NDry Hardener. This greater amount of 2-EHGE likely facilitates a faster and more uniform distribution of the curing agent within the resin, resulting in a quicker curing time. While the hardeners contained more VOC than the resin, the resin contained orders of magnitude higher loadings (max. 15,000,000 μg/g) of 2-EHGE, BPA, and BADGE than the hardeners (max. 3,000 μg/g). The resin SDS did not declare 2-EHGE, and the hardener SDSs did not declare either 2-EHGE or BPA as ingredients (Table S4). BPA and BADGE are SVOCs.^58^

Numerous tentatively identified compounds (TICs) were found in the raw materials, and many lack prior scrutiny of their drinking water toxicity, aesthetic, and secondary impacts (i.e., disinfectant byproducts, microbial growth potential). The hardeners contained the greatest number of TICs, followed by the resin: NDry hardener (33), and QDry hardener (27), resin (14) (Table S5). Of the TICs found, 27 were categorized as VOCs and 24 were categorized as SVOCs (Table S6). Of the chemicals identified that were only present in a composite and not present in either the resin or hardeners, 2-phenyl cyclohexanol and 1-methyl-4-(1-methylethenyl) cyclohexanol were detected in the NDry composite, and benzaldehyde and 2-chlorocyclohexanol were detected in the QDry composite. Some TICs have been found previously by others inside epoxy coatings used for potable water applications or were detected to leach into water from epoxy coatings. For example, the reactive diluent TIC-Benzyl alcohol was previously found in an epoxy^58^ and had leached into drinking water from an epoxy lining.^38^ Only the TIC phenol had either a U.S. federal drinking water maximum contaminant level or healthy advisory level.^57^

### 3.2 Composites Contained Leachable BPA, Unreacted BADGE Monomer, and the 2-EHGE Viscosity Reducer

**Figure 2:**
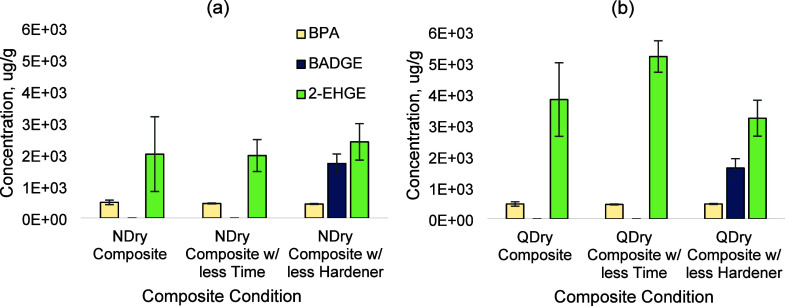
The shortened curing duration did not impact monomer or viscosity reducer loading in either the new (a) NDry composites or (b) QDry composites, but the condition where less hardener was used prompted significantly more monomer to remain in the new composites. Mean and standard deviation values are shown for three replicates.

**Figure 3:**
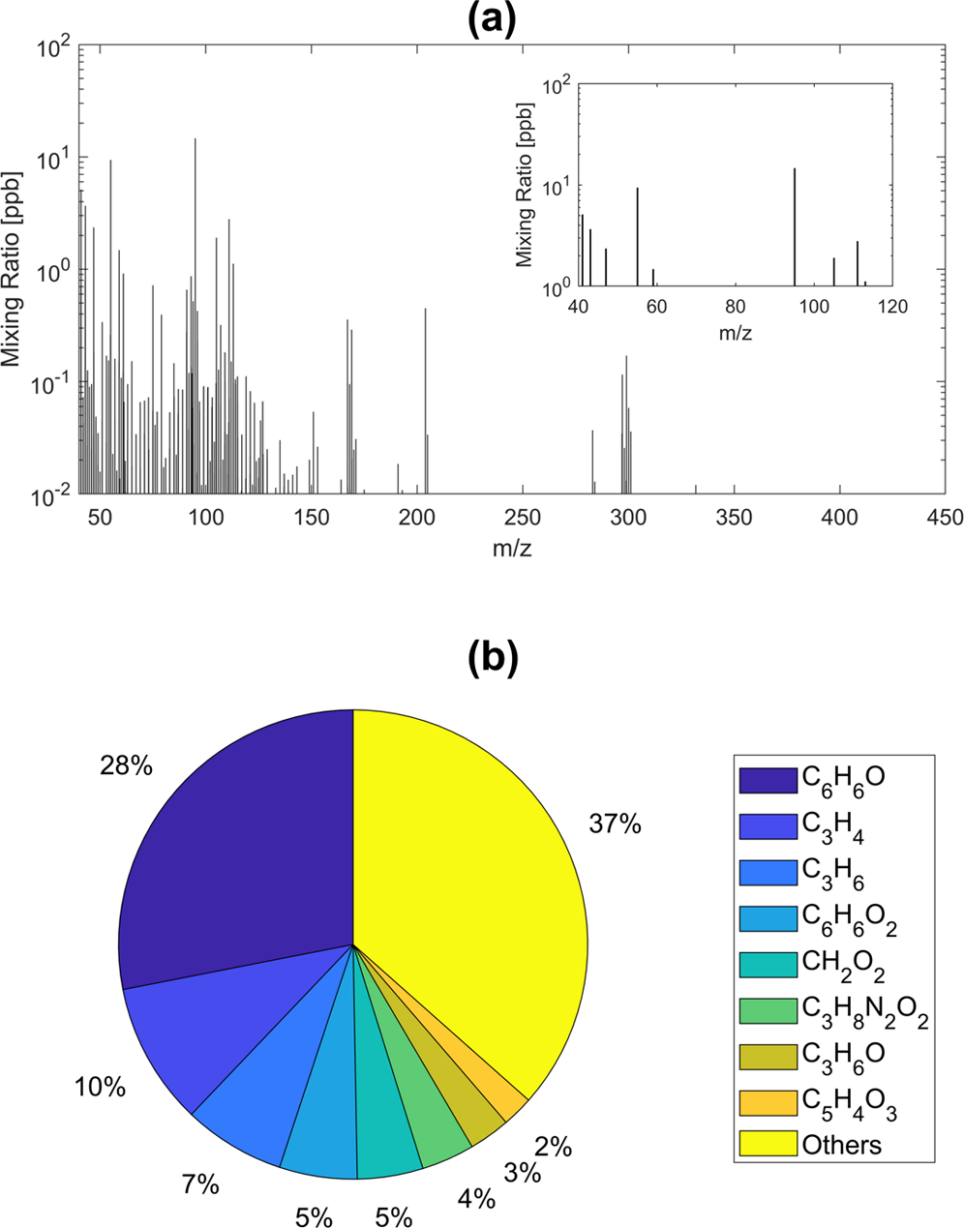
(a) The net increase of the PTR-TOF-MS mass spectrum from epoxy CIPP composite headspace analysis is shown. The top eight identified ions are highlighted in the embedded mass spectrum. (b) Relative abundances of detected parent/fragment compounds for the top eight identified ions (mixing ratio > 1 ppb) are listed with percentage contribution. “Others” represents the sum of all signals with mixing ratios of <1 ppb. The percentage is calculated based on the average mixing ratio during the sampling period.

Manufacturing conditions for both NDry and QDry composites influenced the amount of BADGE remaining in both composites and the amount of 2-EHGE remaining only in the QDry composite. When standard composites were manufactured, BADGE was not detected but 2-EHGE and BPA were identified. When the 5% less hardener was used, BADGE loading inside both composites was significantly greater (*p* = 0.01). This finding indicates that the hardener limitation is a critical factor in the BADGE consumption. However, for this condition, BPA and 2-EHGE loadings were not significantly different from composites that were created using the manufacturer’s explicit instructions. Across all manufacturing conditions, the 2-EHGE loading was greatest for the QDry composites by about a factor of 2 (Figure 2). This result is reasonable as the hardener for QDry composites contained about four times greater initial loading of 2-EHGE than the hardener used for the NDry composites (Figure 3). Several TICs were found in manufacturer’s recommended NDry composites (9) and QDry composites (17). Of those TICs, 11 were categorized as VOCs and 6 were SVOCs (Table S11). As expected, the majority of the initial 2-EHGE, BPA, and BADGE present in the raw materials (90.7 to 100%) was not found in extracts from the composites (Table S7). Overall, both composites contained volatile material as determined by TGA, but the overall magnitudes were not significantly different (*p* = 0.90): NDry composite (3.26 ± 1.41 wt%) and QDry composite (4.81 ± 1.34 wt%).

### 3.3 VOCs in the New Composites Volatilized Into the Air

The PID air monitoring results indicated that both composites released VOCs into the air (Figure S2). Based on this discovery, PTR-TOF-MS analysis was conducted and confirmed that VOCs were released by the new composites into air (Figure 3,Table 2). In the case of all ions being parent ions, the most abundant ion detected was (C_6_H_6_O)H^+^ at *m*/*z* 95.04 which is possibly phenol,^59^ and is a TIC identified by the GC-MS method. Phenol contributed 28% of the total VOC emissions. An unknown *m*/*z* detected at 55.04 is not presented in Figure 3 and Table 2 as it is possibly a water cluster ion that consists of a H_3_O^+^ and two H_2_O (H_3_O^+^(H_2_O)_2_).^60^ Besides (C_6_H_6_O)H^+^, C_3_H_5_^+^ detected at *m*/*z* 41 is possibly propyne;^59^ C_3_H_7_^+^ detected at *m*/*z* 43 is possibly propene or cyclopropane;^59,65^ (C_6_H_6_O_2_)H^+^ detected at *m*/*z* 111 is possibly hydroxy phenol;^89^ (CH_2_O_2_)H^+^ detected at *m*/*z* 47 is possibly formic acid;^90^ (C_3_H_6_O)H^+^ detected at *m*/*z* 59 is possibly acetone.^91^ (C_3_H_8_N_2_O_2_)H^+^ detected at *m*/*z* 105 and (C_5_H_4_O_3_)H^+^ detected at *m*/*z* 113 were unidentified ions.

**Table 2:** Summary of the Top Eight Identified Ions for the QDry Composite

**parent or fragment ions**	*m***/***z*	**chemical formula**	**mixing ratio [ppb]**	**mol. wt.** [g/mol]
(C_6_H_6_O)H^+^	95.042	C_6_H_6_O	14.646	94.11
C_3_H_5_^+^	41.040	C_3_H_4_	5.118	40.06
C_3_H_7_^+^	43.055	C_3_H_6_	3.668	42.08
(C_6_H_6_O_2_)H^+^	111.043	C_6_H_6_O_2_	2.796	110.11
(CH_2_O_2_)H^+^	47.014	CH_2_O_2_	2.362	46.03
(C_3_H_8_N_2_O_2_)H^+^	105.064	C_3_H_8_N_2_O_2_	1.909	104.11
(C_3_H_6_O)H^+^	59.049	C_3_H_6_O	1.48	58.08
(C_5_H_4_O_3_)H^+^	113.023	C_5_H_4_O_3_	1.120	112.08

According to Table 1, CIPP ingredients included a variety of alcohols and aldehydes that are denser in mass, but the *m*/*z* for the top eight detected ions ranged from only 40 to 114. This is because protonated compounds can be fragmented into stable fragments with the reduction of the electric field in the PTR-TOF-MS drift tube.^62−64^ C_3_H_5_^+^, C_3_H_7_^+^, (C_6_H_6_O_2_)H^+^, (CH_2_O_2_)H^+^, (C_3_H_6_O)H^+^, and (C_5_H_4_O_3_)H^+^ are possibly fragment ions of the emitted alcohols and aldehydes. The fragmentation for a variety of alcohols was found to include *m*/*z* 41, *m*/*z* 43, *m*/*z* 47, *m*/*z* 111, and *m*/*z* 113, while the fragments for aldehydes were *m*/*z* 41, *m*/*z* 59, and*m*/*z* 111.^61,63,66^ (C_3_H_8_N_2_O_2_)H^+^ detected at *m*/*z* 105.06 is possibly the fragment of the nitrogen-containing raw component C_13_H_26_N_4_O_2_.

### 3.4 Chemicals Found in Drinking Water Exceeded Some Health Limits

All composites leached organic carbon compounds into drinking water after each leaching period, and TOC concentrations ranged from 0.26 to 2.33 mg/L (Figure S5). The initial high TOC concentration standard deviation values for the first leaching period suggested potential nonuniform chemical loading on composite surfaces. When TOC results were compared to the chemical mass quantified using the GC-MS method, less than 0.1% of the total organic compound mass was chemically identified by the authors. Therefore, 99.9% of the organic material released to the drinking water by the composites was not chemically identified.

**Figure 4:**
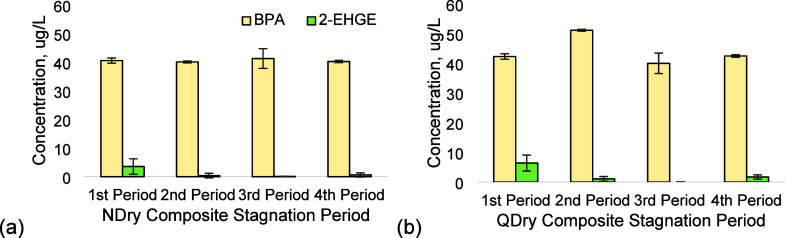
BPA was consistently detected at the highest concentration for both (A) NDry and (b) QDry composites compared to the 2-EHGE when CIPPs were exposed to drinking water. BADGE was not detected in drinking water after both composites were exposed to water. Mean and standard deviation values are shown for three replicates.

For the three chemicals studied, all of the composites leached 2-EHGE and BPA during all four stagnation periods (Figure 4). These compounds were also found inside the new composites at notable loadings. No significant difference was found between the BPA aqueous concentrations for both composites during all four stagnation periods (*p* = 0.900). A significant difference was observed for 2-EHGE concentrations comparing the first period of stagnation with the subsequent two periods (*p* = 0.020). The final 96 h of leaching period confirmed that 2-EHGE and BPA were still leaching from the composites. Changes in manufacturing conditions did not significantly change the aqueous concentration of either of these compounds (Figure S6). BADGE was not detected in drinking water during any leaching period.

To estimate human exposure for full-scale drinking water pipes, results were extrapolated using the surface area to water volume ratio (Table 3). This practice is used for ANSI/NSFI Standard 61 material certification to estimate field-scale chemical concentrations. Because a standard exists for the use of 4 in. or larger diameter CIPPs,^5^ these pipe sizes were the primary focus of the author’s analysis. First, BPA leaching results for NDry and QDry composites here (1,228 and 1341 ug/m^2^ day, respectively) were within levels reported in the literature for epoxy coatings used for drinking water materials (0.975 to 6,935 ug/m^2^ day) (Table SI-3). Therefore, the results here are reasonable. Second, as pipe diameter increased from 4 in. to 36 in., as expected, the predicted contaminant concentration observed decreased^5^. Both NDry and QDry CIPPs were predicted to cause BPA to exceed the 20 μg/L Minnesota short-term drinking water limit.^9^ Predicted BPA concentrations for all diameters examined exceeded the more stringent World Health Organization and European Union BPA drinking water limits by 30x to 15x, respectively. Should epoxy CIPP be used for pipes smaller than 4 in. diameter, BPA levels would be predicted to exceed not only the Minnesota drinking water BPA limit but industry BPA limits too (Table S9). No federal or state drinking water limits (regulatory or guidance) were found for 2-EHGE. Industry 2-EHGE exposure levels ranged from 0.3 to 10 μg/L depending on exposure duration. Because a prior CIPP 2-EHGE leaching report indicated “>300 μg/L 2- EHGE”^2^ was found, more publicly available data is needed.

**Table 3:** Leaching Results Were Scaled to Different Diameter Water Mains by Considering the CIPP Surface Area to Water Volume Ratio^a^

		**Predicted concentration for the 1st (3rd) and 4th stagnation period and water main pipe diameter, inches**					**Drinking Water Health-Based Exposure Limits**			
**compound and composite type**		**36**	**24**	**12**	**6**	**4**	**WHO**	**EU**(67)	**MN (Short-term, Long-term)**(68)	**Stnd 61 (SPAC/ /TAC/ STEL)**(69)
BPA	NDry	3 - 3 - 3	5 - 5 - 5	10 - 10 - 10	20 - 20 - 20	30 - 30 - 30	1	2.5	100/20	10/100/200
	QDry	3 - 3 - 3	5 - 5 - 5	10 - 10 - 10	21 - 20 - 21	31 - 29 - 31				
2-EHGE	NDry	<- < - <	<- < - <	1 - < - <	2 - < - <	3 - < - <			–/–	0.3/3/10
	QDry	1 - < - <	1 - < - <	2 - < - <	3 - < - 1	5 - < - 1				
TOC	NDry	<- < - <	<- < - <	<- < - <	1 - < - <	1 - < - <				
	QDry	1 - < - <	1 - < - <	2 - < - <	3 - < - <	5 - < - <				

a BADGE was not detected during any leaching period; “<” indicates contaminant was not detected; all results are reported as μg/L; concentrations were compared against WHO, EU, State of Minnesota short- and long-term/ANSI/NSFI Standard 61 thresholds. WHO = World Health Organization drinking water guideline; EU = European Union limit for water intended for human consumption; State of Minnesota drinking water guidance values for chronic exposure and for short-term exposure; for the industry product testing procedure ANSI/NSFI Standard 61: SPAC = maximum recommended concentration by industry of a contaminant in drinking water that a single product may contribute; TAC = maximum concentration of an unregulated contaminant that is recommended for a public drinking water supply by industry; STEL = maximum concentration of a contaminant that is recommended in drinking water for an acute exposure by industry.

**Figure 5:**
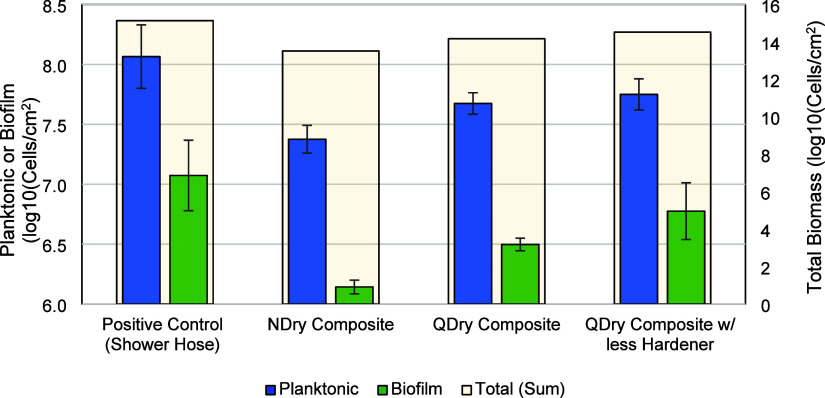
Planktonic and biofilm production (primary left axis) from three different composite types compared to a positive control of a shower hose, normalized to surface area of material. Total biomass produced per cm^2^ of material (right secondary axis) was calculated by summing planktonic and biofilm cell concentrations.

Three formulations were tested for biological growth potential, including NDry, QDry, and QDry with 5% less hardener. These were compared to a shower hose, a positive control that leaches a significant amount of carbon compounds that contribute to unwanted growth.^70^ Although each of the formulations had differences in the types of carbon compounds released, these did not have a strong impact on the biomass production. Biomass was produced on all composite formulations. While the NDry composite did have slightly less biomass production than QDry, there was little difference between QDry and QDry with less hardener, despite the differences in BADGE production noted above. This likely indicates that the biodegradable carbon released from all formulations is similar. It should be noted that the surface area to volume ratio is often higher in shower hoses than in distribution pipes where CIPP may be used, and thus, this contribution to biomass production may have a more limited impact on the water than shower hoses (Figure 5).

## 4 Implications

Given that 43% of U.S. water mains are greater than 8 in. diameter, CIPPs could become ubiquitous as a drinking water contact material (Table 1, Table S9). Results from the present study show that VOCs were present in the new CIPPs, which volatilized into the air and leached into drinking water. About 99.9% of the chemical mass leached into drinking water was not identified. BPA exceeded the State of Minnesota, EU, and WHO drinking water limits. However, nearly all of the chemicals released from the CIPPs did not have regulated drinking water standards or health advisory levels.

Because publicly available information is limited and considering the findings of the present study, flushing, and chemical testing are recommended before a new CIPP is returned to service. Larger diameter CIPPs should theoretically prompt lower chemical concentrations in drinking water compared to smaller diameter CIPPs. However, the full array and magnitude of chemicals leached are not yet known. Chemicals and concentrations in actual water distribution systems may differ across resins, curing conditions, and even site-specific conditions. Caution while interpreting drinking water, chemical analysis results should be applied as disinfectant reactions (i.e., BPA),^37^ hydrolysis reactions (i.e., BADGE),^32^ and water temperature^26^ can influence the degree epoxy-related chemicals are detected. A worst-case stagnation period for the CIPP should be considered to allow chemicals to accumulate in water, so that laboratories can detect them.

Identifying chemicals leached from CIPPs and their significance to health or secondary water quality impacts (i.e., DBP formation, biofilm growth) remains challenging due to analytical limitations, the lack of prior studies, and public information sharing by industry. Standard drinking water quality analysis methods (USEPA Method 524.2,^67^ USEPA Method 8270E)^68^ used in past field drinking water CIPP studies would not detect 41 of 51 chemicals found in the present work (Table S11). Second, the SDSs did not describe all chemicals in the raw materials that leached into drinking water from the composites (i.e., BPA, 2-EHGE). Third, because product certification results are not publicly disclosed, health officials are prevented from understanding which chemicals are created during manufacture, remain as intermediates, and can leach out. Field studies may help to better understand CIPP drinking water quality impacts, but result interpretation will be challenging due to the lack of a bench-scale understanding of the materials. Characterizing the wastewater and condensate generated during steam and hot water drinking water CIPP curing operations may help expedite knowledge about chemicals that are produced during manufacture and may leach out from the new CIPP.

There are several limitations associated with this study, and the results provide a basis for future investigations. The lack of prior epoxy CIPP field and laboratory testing in the literature inhibited a greater comparison of the study results. Many CIPP manufacturers declined to provide the authors with material SDSs for products they had already installed and were actively installing. The analytical methods applied here could not characterize all contaminants leached, as 99.9% of the organic mass leached from the epoxies was not chemically identified. The authors could not review ANSI/NSFI standard 61 testing results for the products examined or other CIPPs because the results are not publicly available.

**Figure 6:**
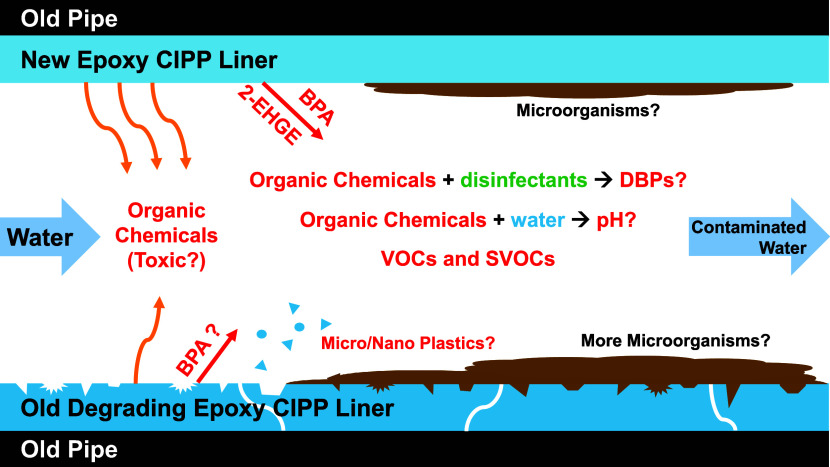
Water quality impacts of drinking water CIPPs require additional scrutiny. Organic chemicals include VOCs and SVOCs and those analyzed in this study. DBP represents disinfection by products.

The long-term impacts of these materials on drinking water quality remain unknown; however, a conceptual model of the short- and long-term water quality performance of CIPPs was developed (Figure 6). Unidentified VOCs and SVOCs in the present study may be more hydrophilic requiring different analytical methods for their identification and quantification. Chemical differences across drinking water CIPP resin and hardener batches from the same and different manufacturers remain unclear. These differences may influence chemical leaching. Study results do not describe the water quality impacts of aged and degraded epoxy.

While some drinking water CIPPs use a plastic film between the CIPP epoxy and drinking water during curing, the role of the film upon leaching was not examined here. Though this practice was reportedly preventing leaching in CIPP sewer installations, it was subsequently found that the heating process caused the plastic film to melt and degrade, thereby enabling direct water contact with the resin.^69^ The degree to which CIPP resin manufacturers and CIPP contractors inadvertently chemically contaminate the raw materials and the new CIPP during an installation also remains unclear. CIPP resin and new CIPP chemical contamination problems were discovered by monitoring sewer CIPP installations previously.^51,70^

An important discovery here was that some industry claims about drinking water CIPP were not supported. One claim was that epoxy CIPP products had no VOCs.^75^ Here, VOCs were found in the raw materials and composites, and PID and PTR-TOF-MS results revealed that VOCs volatilized from the new epoxy into the air. While all VOCs emitted into air were not identified, this discovery merits further examination. Like sewer CIPP technology, there may be worker safety, public safety, and environmental implications^51,52,71,72^ of drinking water CIPP chemical emissions into air during and after manufacture. For decades, steam CIPP waste discharged into the air from sewer CIPPs was reported to be “harmless” but was instead found to be a harmful complex mixture of VOCs, SVOCs, solvents, liquid droplets, microplastics, and nanoplastics. Epoxy CIPP is known to pose a dermal risk to workers,^73^ but no air pollution studies were found. Drinking water CIPP wastewater and condensate investigations are recommended to characterize the chemical composition, magnitudes, toxicity, and resulting environmental, human health, and wastewater treatment facility impacts, if any.^73−75^ A better understanding of how drinking water biofilms interact with new and old CIPPs and potential release of microplastics and nanoplastics is recommended.

As drinking water infrastructure contact materials transition from metal and concrete to plastic, a better understanding of their drinking water quality impacts is recommended. Historically, U.S. drinking water providers have adopted new plastic technologies and subsequently discovered avoidable reliability and chemical water safety issues.^76−82^ In some cases, this has required the subsequent removal of these plastic assets. Outside the U.S., allegedly a certified CIPP drinking water product was associated with contamination.^83^ Within buildings, plastic water technologies also have encountered similar mechanical and drinking water performance challenges.^84−88^ Because many U.S. drinking water pipes are expected to be repaired in the coming years, this topic is important. Health officials should identify and resolve the knowledge gaps. At present, the limited information available about these materials has shifted the burden of potential health and financial costs to the users.

## Supporting Information Available

Additional background material, method descriptions, figures, and tables (PDF)
