# Supplementary material for: Release of Bisphenol A and Other Volatile Chemicals from New Epoxy Drinking Water Pipe Liners: The Role of Manufacturing Conditions
Source: Environ Sci Technol. 2025 Jan 2;59(1):767–78. doi: 10.1021/acs.est.4c08663 (PMC11741100; doi:10.1021/acs.est.4c08663)
Supplement: Supplementary file 1 — es4c08663_si_001.pdf [file es4c08663_si_001.pdf]

Release of bisphenol A and other volatile chemicals from new epoxy drinking water pipe liners: The role of manufacturing conditions

Pritee Pahari, Samuel Spears, Jianghui Liu, Sydney Butler, Shantanu Sonowane, Anthony Garcia, Madeline Larsen, Caitlin R. Proctor, John A. Howarter, Jeffery Paul Youngblood, Nusrat Jung, Andrew J. Whelton

Corresponding author: Andrew J. Whelton, Lyles School of Civil Engineering, Division of Environmental and Ecological Engineering, Purdue University, 550 Stadium Mall Drive, West Lafayette, IN 47907, awhelton@purdue.edu

Summary: 20 pages, 6 figures, 6 tables

Table of Contents

SI-Introduction 3

Figure SI-1. The drinking water from East Bay Municipal District (Oakland, California) placed in contact with the new epoxy CIPP water main became milky white and foamy 3

Table SI-1. U.S. and Canada epoxy drinking water applications 3

Table SI-2. Epoxy studies from outside the U.S. and Canada 5

Table SI-3. Normalized bisphenol-A BPA leaching rates from worldwide studies 6

SI-Materials and Methods 7

Table SI-4. Manufacturer recommended resin: hardener mixing ratio, curing time, curing temperature and list of ingredients in the SDS for the resin and hardeners 7

Method. Preparation of resin hardener mixtures 7

Method. Preparation of synthetic Eastern U.S. drinking water 8

Method. PID air monitoring 8

Method. PTR-TOF-MS air monitoring 8

Figure SI-2. Experimental setup for epoxy CIPP composite headspace analysis. 9

Figure SI-3. Experimental sequence for Epoxy CIPP headspace analysis. 9

Figure SI-4. The schematic diagram represents the step-by-step procedure during curing of a composite. 11

Method. Biological growth assessment 11

SI-Results and Discussion 11

Figure SI-5. Thermogravimetric analysis of the resin, hardeners, and the composites revealed the presence of a notable amount of volatile material. 12
Figure SI-4. VOCs were emitted from NDry and QDry Composites into the air inside a closed container after sitting for 24 h and and PID signal was measured once the container was opened 13
Figure SI-5. The drinking water TOC levels were examined after each exposure period for NDry and QDry composites. The first, second, and third periods were 24 h duration. The fourth period was 96 h in duration. 14
Figure SI-6. The first 24 h leachate concentration and trend for the NDry and QDry composite with less time and less hardener were like that of the NDry and QDry Composite. 14
Table SI-6. The presence of BPA, BADGE, and 2-EHGE in each resin, hardeners and composites in different manufacturing conditions were summarized 14
Table SI-7. The wt% of 2-EHGE, BPA, and BADGE extractable from the composites was sometimes influenced by manufacturing conditions. 15
Table SI-8. The wt% of organic compound residual leached into the water and compounds remaining in the composite during the study period 15
Table SI-5. Several TICs were detected in the resin, hardeners, and composites 15
Table SI-6. TICS detected across materials 16
SI-References 19

SI-Introduction

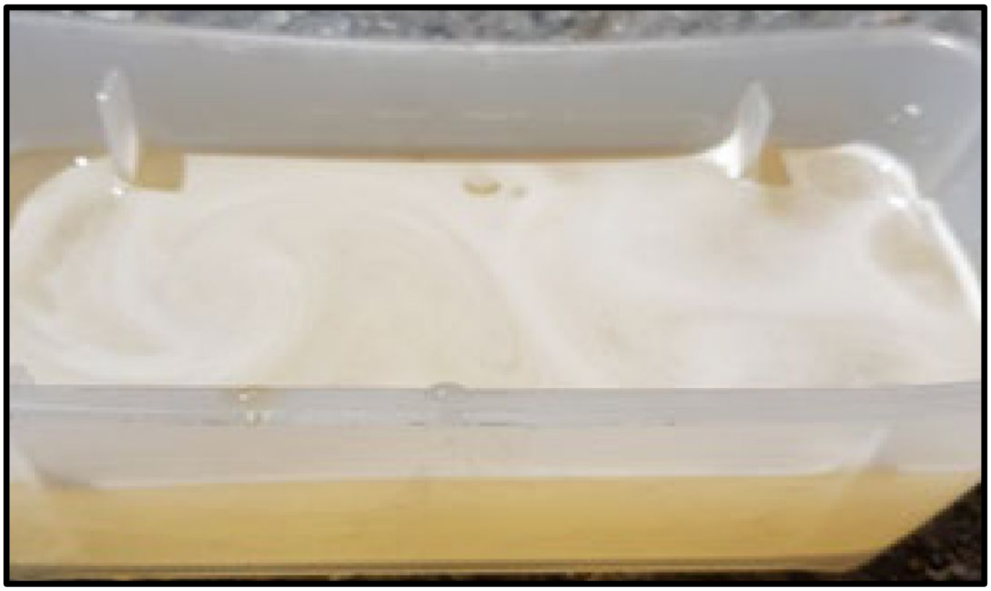

Figure SI-1. The drinking water from East Bay Municipal District (Oakland, California) placed in contact with the new epoxy CIPP water main became milky white and foamy

Table SI-1. U.S. and Canada epoxy drinking water applications

Year	Epoxy Test Condition	Water Quality Impacts
2017 (Randtke et al., 2017)	1 NSFI approved formulation	1. BADGE ranged from 13-340 µg/L
	Water type: lab prepared water (pH 8, 6.5)	2. BADGE, BFDGE hydrolyzed and decayed
	Disinfectant: Free chlorine, Monochloramine	3. BPA, BPF did not decay
	Temperature: 20-25 ^{o}C	4. BPA, BPF, and triethylenetetramine (TETA) reacted with both disinfectants
	Duration: 10 days	5. BADGE did not react with disinfectants
2010 (Deb et al., 2010)	1 NSFI approved formulation (new)	1. TOC up to 6.3 mg/L on day 1, up to 1.7 mg/L on day 30
	Water type: lab prepared water (pH 8)	2. BPA 33 µg/L (day 1), trace (day 30)
	Disinfectant: Free chlorine, Monochloramine	3. Odor described as “plastic/ chemical/ sweet”
	Temperature: 19-23 ^{o}C	4. Disinfectant loss occurred
	Duration: 30 days	5. DBPs were produced (THM <60 µg/L & HAA5< 80 µg/L)
	1 NSFI approved formulation (5 years old)	1. TOC stable between 1.6 and 3.5 mg/L over 30 days
	Water type: lab prepared water (pH 8)	2. Disinfectant loss occurred
	Disinfectant: Free chlorine	3. DBPs were produced (HAA5 < 12 µg/L)
	Temperature: 19-23 ^{o}C	4. Microbial growth occurred
	Duration: 30 days	
2007 (Heim & Dietrich, 2007)	1 formulation	1. TOC 0.6 mg/L
	Water type: lab prepared water (pH 7.7-7.9)	2. Odor described as "plastic/adhesive/putty"
	Disinfectant: Free chlorine, Monochloramine	3. BPA; phenol, nonyl-phenol, styrene, toluene, benzaldehyde
	Room temperature	4. DBPs were produced (3-9 µg/LTHM)
	Duration: several months	
2002 (Satchwill, 2002)	5 NSFI approved formulations	1. Total BTEX ranged from 0.2-48 mg/L
	Water type not reported	2. TOC ranged from 34-345 mg/L
	Temperature: 49 ^{o}C	3. Pre-rinsing had little effect on leaching
	Duration: 72 hr	4. Extended dry times had limited impact on reducing leaching
1989 (Albenet al., 1989)	1 formulation in lab; 3 field storage tanks	1. MIBK, *o*-, *m*-, *p*-xylene; ethoxy ethyl acetate; methyl benzaldehyde
	Water type: lab prepared water (pH 8.5)	2. Disinfectant loss occurred
	Disinfectant: Free chlorine	3. DBPs may have been produced
	Temperature: 23 ^{o}C	4. Water soaking caused more rapid leaching than air drying
	Duration: 4 weeks	5. MIBK and xylenes detected in two of the three tanks

Table SI-2. Epoxy studies from outside the U.S. and Canada

Year	Epoxy Test Condition	Water Quality Impacts
2016, Finland (Rajasärkkä et al., 2016)	Six 2-9 year old linings in the field	1. BPF, 4-NP, 4-*t*-octylphenol rarely found; trace concentrations<br>2. BPA was detected in most samples; Maximum (cold water) 0.25 μg/L and (hot water) 23.5 μg/L<br>3. Older epoxy leached 4-20x more BPA than newer epoxy
2014, France (Bruchet et al., 2014)	3 linings<br>Water type: mineral water (pH unknown)<br>Disinfectant<br>Temperature: 20 °C<br>Duration: 6 months	1. Epoxy #3 showed increasing leaching during 5 months<br>2. BPA only found in absence of disinfectant, no BPF found at all<br>3. 2,4,6-trichlorophenol (TCP), a BPA chlorination by-product sporadically observed in the chlorinated water<br>4. Discontinuation of disinfectants enhanced leaching of BPA and TCP
	27 old coated water tanks, 200 old coated pipe sections (10-20 years old)	1.TANKS: No BPA, BPF or TCP<br>2. PIPES: High frequency of BPA and BPF detection, sometimes with maximum values around 1 μg/L; TCP and 2,4,6-trichloroanisole were found in a few samples
2012, Japan (Kosaka et al., 2012)	2 linings<br>Water type: tap water (pH unknown) and lab prepared water (7.0)<br>Disinfectant: Chlorine (tap water only)<br>Temperature: 23 °C<br>Duration: 24 months	1. Levels of BPA, chlorinated BPA increased in retained water<br>2. BPA leaching did not decrease during 24 months of continuous test<br>3. Sums of the levels of BPA and chlorinated BPA had a negative relationship with the residual chlorine levels<br>4. TCP was produced; 12 μg/L (24 months)
2007, England (Jackson et al., 2007)	Five 1-10 years old linings in the field	1. No detectable TOC<br>2. GC-MS analysis found no epoxy specific compounds resulting from 4 epoxies<br>3. One epoxy showed evidence of leaching of 4-*t*-butyl phenol (4-TBP), and the presence of halogenated 4-TBP products, with a max. 2.2 μg/L
2002, Spain (Romero et al., 2002)	5 linings<br>Water type: ultrapure water<br>Temperature: 40-45 °C<br>Duration: 5 days	1. BPA of 0.02-0.03 μg/cm^2<br>2. Benzyl alcohol of up to 180 μg/cm^2<br>3. Phthalates of 0.04-0.3 μg/cm^2, benzaldehyde, 4-NP, ketones, antioxidants
2002, Korea (Bae et al., 2002)	3 linings<br>Water type: DI water, lab prepared water (pH 7.0)<br>Temperature: 20, 50, 75, 100 °C<br>Duration: 6 hr (50-100 °C), 24 hr (20 °C)	1. BPA from unit area of epoxy resin lining was in the range of 10.68 to 1,734 μg/m^2<br>2. Higher risk of BPA leaching to drinking water during a summer season<br>3. Microbial growth was higher with epoxy than in a stainless-steel tank
1999, France (Rigal & Danjou, 1999)	1 lining, 1 hr after installation (test conditions unknown)	1. Benzyl alcohol (345 μg/L), monoglycidyle ether of butane diol (12 μg/L), diglycidyl ether of butane diol (386 μg/L), diaminodiphenylmethane (72 μg/L), and a "series of derivatives from butanediol and diaminodiphenylmethane"<br>2. Total flavor number: 6 "glue and bitter almond" to 2.5 "bitter flavor"

Table SI-3. Normalized bisphenol-A BPA leaching rates from worldwide studies

Locations	Epoxy Details, including ages*	Testing conditions						Max. BPA leaching rate (µg/m^2-day)
		Water Type	Sample Exposure Duration **	SA:V ratio ***	Temp.	pH	Disinfectant	
Product Testing Procedure								
NSF/ANSI 61-2016	New lining materials	lab prepared	72 hr at 23°C, 3 hr at 60 ^{o}C, 3 hr at 82 ^{o}C	50 cm^2/L ß	23°C to 82 ^{o}C	8.0	0 mg/L	-
Literature Reported Epoxy Leaching Methods and Calculated BPA Leaching Rates								
USA (Deb et al., 2010)	1 new Standard 61 approved formulation	lab prepared	24 hr	As 4” diameter pipe	19 to 23 ^{o}C	8.0	2 mg/L free chlorine; 4-6 mg/L monochloramine	838
France (Bruchet et al., 2014)	3 linings, (after 108 d use)	mineral	24 hr	50 cm^2/L	20 ^{o}C	NR	0.5 mg/L free chlorine; 0.25 mg/L chlorine dioxide	18.0
Japan (Kosaka et al., 2012)	1 lining (after 24 months use)	tap	16 hr	0.2 m^2/L	23 ^{o}C	NR	0.38-0.66 mg/L free chlorine	0.975
	1 new lining prepared in lab	lab prepared		0.0546 m^2/L	23 ^{o}C	7.0	0 mg/L	11.55
Korea (Bae et al., 2002)	3 new lining formulations	deionized & lab prepared	6 hr	reported per unit area	100 ^{o}C (50, 75 ^{o}C also tested)	7.0	0 mg/L	6,936
			24 hr		20 ^{o}C			65
Spain (Romero et al., 2002)	5 new epoxy formulations	deionized	5 days	reported per unit area	40 to 45 ^{o}C	NR	0 mg/L	60

Maximum BPA leaching rates were calculated using the maximum concentrations under reported testing conditions, the duration of the leaching test, and the surface area to volume (SA: V) ratio as needed; ß= Minimum SA: V required; NR represents not reported; *Total test time varied, age reported is the age at which the maximum concentration was found. “New” means the concentration was measured the first exposure water. **Duration refers to the stagnation time in the test water, not to the age of epoxy or total length of test; ***The surface area to volume ratio, or information used to calculate it, given in the paper. Some papers reported concentration per unit are

SI-Materials and Methods

Table SI-4. Manufacturer recommended resin: hardener mixing ratio, curing time, curing temperature and list of ingredients in the SDS for the resin and hardeners

Material	Manufacturer Recommended			Total List of Ingredients on SDS
	R:H Ratio	Cure Time	Cure Temp.	
Resin	-	-	-	75-80% Bisphenol-A Epichlorohydrin Epoxy Resin<br>10-20% [[(2-ethylhexyl) oxy] methyl] oxirane<br>1-5% Silicon Dioxide, chemically prepared
NDry Hardener	4:1	3.5 h (3-4)	130°F	>80% polyamides<br><15 % Benzyl Alcohol<br>>5% Isophoronediamine
QDry Hardener	2:1	2 h	130°F	55-65% reaction products with phenol and formaldehyde<br>15-20 % Triethylenetetramine<br>15-20% Phenol

R:H ratio = Resin: hardener ratio

Method. Preparation of resin hardener mixtures

A resin-hardener mixture of 100 g was prepared for each batch of composites. Two different size composites were manufactured using the same formulations (size 1: 10.16 cm x 10.16 cm x 0.6 cm; size 2: 5.08 cm x 15.08 cm x 0.6 cm). The composite thickness was similar to the wall thickness of CIPP water mains. The larger composites were used for solid-liquid extraction and the smaller composites were used to examine chemical leaching into drinking water. The resin-hardener-felt material underwent thermal curing (dry air) at the manufacture's recommended temperature of 54.4 ^{o}C and time (3.5 h and 2 h for NDry Hardener and QDry Hardener, respectively) in an oven (Model 20GCE, Hogentogler and Company, Inc., USA). Composites were cured on a 25.4 cm x 25.4 cm x 0.16 cm stainless steel plate covered with perforated Teflon® film.

Method. Preparation of synthetic Eastern U.S. drinking water

The water composition was formulated to replicate the mineral composition found in a typical drinking water. Nano-pure water with a resistivity of 18.2 MΩ cm was used as a base. The water chemical composition consisted of 0.8045 g of $MgSO_4 . 7\ H_2O$, 0.0070 g of $Al_2(SO_4)_3 \cdot 18H_2O$, 0.0970 g of KNO_3, 0.5680 g of $NaHCO_3$, 0.7544 g of $NaSiO_3$, 0.2530 g of $CaSO_4 \cdot 2H_2O$, 0.2050 g of $CaCl_2 \cdot 2H_2O$ and 0.0009 g of Na_2HPO_4 in 10 L of Type I water.

Method. PID air monitoring

The PID was calibrated with 10 ppm isobutylene (correction factor of 1) and zeroed using ambient air. After 24 h of the new composite The PID monitored every second, and results were averaged by the device over and recorded every 15 seconds. The PID value reported was recorded. After composites were manufactured, they were placed in sealed glass jars with PTFE caps. After 24 h, the jar was opened and the PID inlet was placed within the jar. The lid was placed back above the PID inlet to reduce VOCs from escaping. PID measurements were conducted for 5 min. This process was repeated for each jar; recalibration was performed if the device did not return to 0 ppm after removal from most recent jar.

Method. PTR-TOF-MS air monitoring

The headspace analysis was carried out in three steps: purge, equilibrium, and sampling using the setup described in **Figure SI-2**. For purging, a compressed zero-air cylinder (Indiana Oxygen Company, Indianapolis, IN, U.S.) was adopted, the pressure of zero-air was set to be 90 psi and the flow rate of zero-air was controlled by a mass flow controller at approximately 1.0 L min^{-1}. A three-way tee was connected to the zero-air pipeline to allow excess flow followed by a hydrocarbon trap (Model 2-2445-U, Supelco Inc., Bellefonte, PA, U.S.) to remove impurities in zero-air. A solenoid valve (Parker, Mayfield Heights, OH, U.S.) was used to control zero-gas flow into the vial and this valve was controlled by a PTR-TOF-MS auto system. For sampling, a three-way hand valve (McMaster-Carr, Elmhurst, IL, U.S.) was used. The experimental sequence for the headspace experiment is illustrated in **Figure SI-3**.

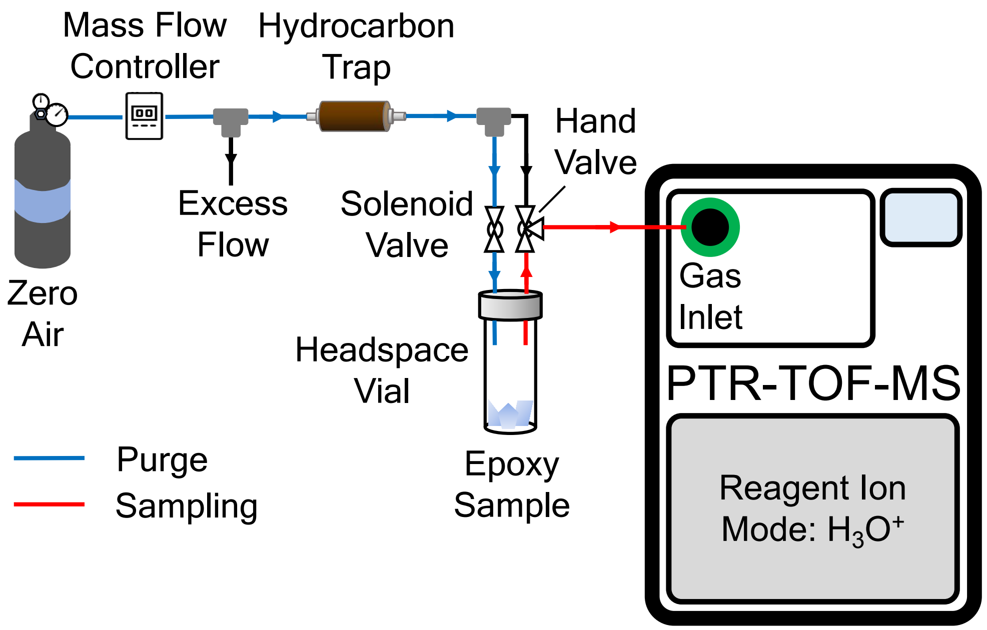

Figure SI-2. Experimental setup for epoxy CIPP composite headspace analysis.

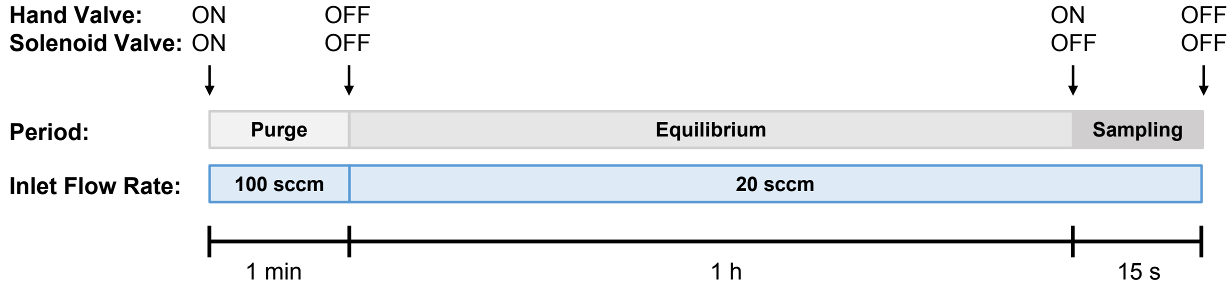

Figure SI-3. Experimental sequence for Epoxy CIPP headspace analysis.

To conduct sample purging, both the hand valve and solenoid valve was turned on for 1 min with the PTR-TOF-MS inlet sampling rate set at 100 sccm to allow zero-air to enter the vial and purge out the gas in the vial. Both valves were turned off immediately after 1 min of purging, after a purging period the vial was rested for a 1 h to reach new equilibrium with zero-air. The inlet sampling rate was set to 20 sccm after purging to accommodate the volume of the vail and provide adequate air sample to the PTR-TOF-MS inlet. After 1 h of equilibrium, hand valve was used to allow 15s of sampling period. The sampling frequency occurred at 1 Hz using hydronium (H_3O^+) as the reagent ion. The ionized molecules ($VOCH^+$) with a mass-to-charge ratio (*m/z)* from 40 to 450 were

recorded. The ionization field energy (E/N) was maintained at approximately 104 Td with the operational pressure, voltage, and temperature of the drift tube set at 2.8 mbar, 500 V and 120 °C, respectively. The PTR-TOF-MS was calibrated with a gas standard that contained approximately 2 ppm of 16 different compounds. For VOCs not available in the gas standards, the mixing ratios of these compounds were calculated based on a proton transfer theory.[14,15] The transmission curves were determined by PTR-MS Viewer (Ionicon Analytik Ges.m.b.H., Innsbruck, Austria) using the daily calibration signals and the headspace signals were analyzed using Ionicon Data Analyser (Ionicon Analytik Ges.m.b.H., Innsbruck, Austria). Additional information regarding the PTR-TOF-MS calibration, raw signal/mixing ratio conversion and data analysis can be found elsewhere.[16,17] In addition, three empty vials were measured with the same procedures to obtain baseline signals for background correction. The net increased PTR-TOF-MS mass spectrum was determined by subtracting the average baseline signals from the average sampling signals.

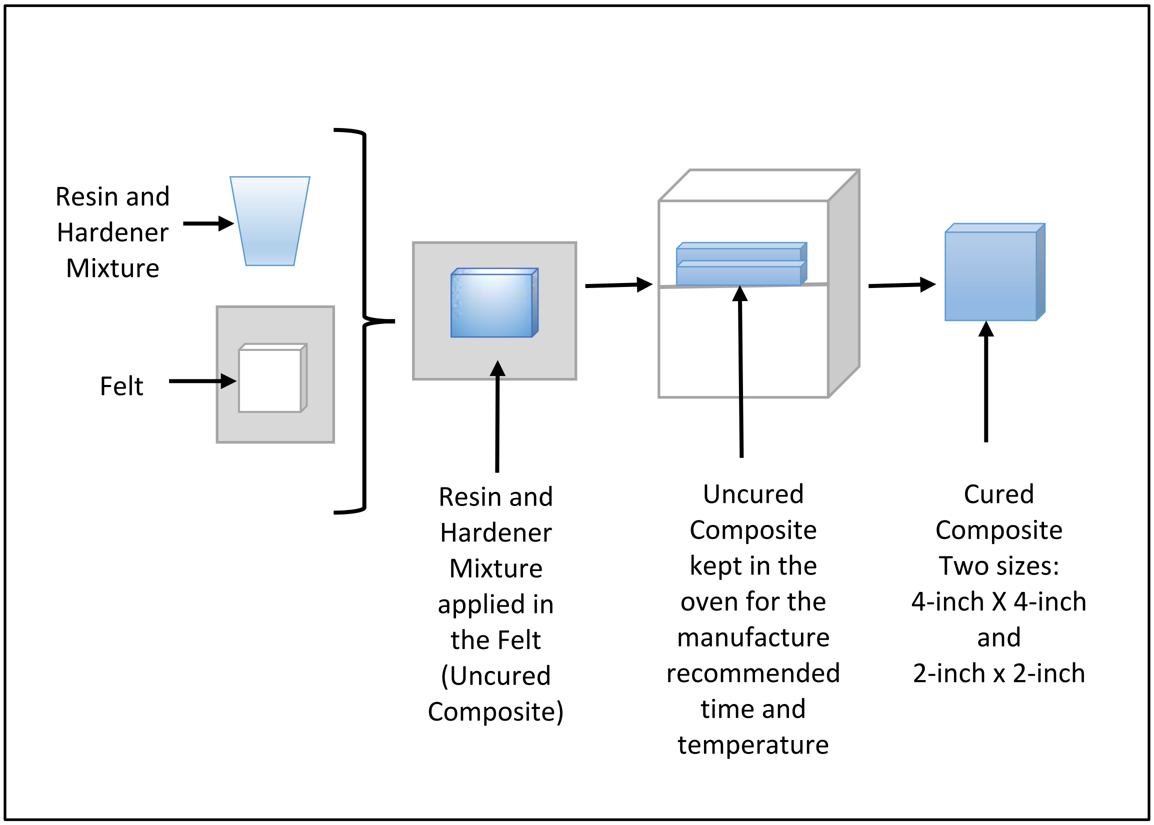

Figure SI-4. The schematic diagram represents the step-by-step procedure during curing of a composite.

Method. Biological growth assessment

Pieces of composite, prepared as above, were incubated in triplicate at 30 °C for 14 days in muffle oven sterilized jars to measure growth both in water (planktonic) and attached to the material (biofilm). Composites were incubated with Evian® bottled water, representing a standardized drinking water community, and a minimal media mix that provided all nutrients other than carbon, as drinking water is often carbon limited. This test provides insights into how pipe material can provide growth substrate and contribute to unwanted biological growth.

SI-Results and Discussion

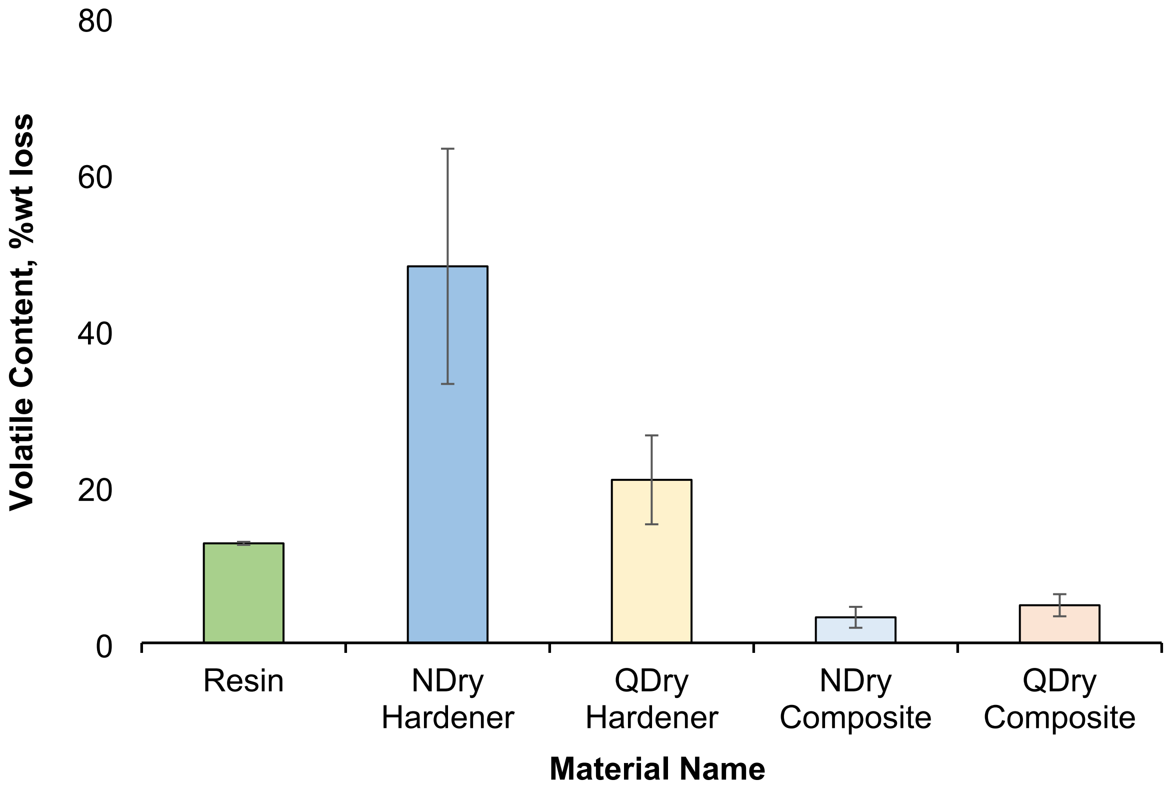

Figure SI-5. Thermogravimetric analysis of the resin, hardeners, and the composites revealed the presence of a notable amount of volatile material. Mean and standard deviation values are shown for three replicates.

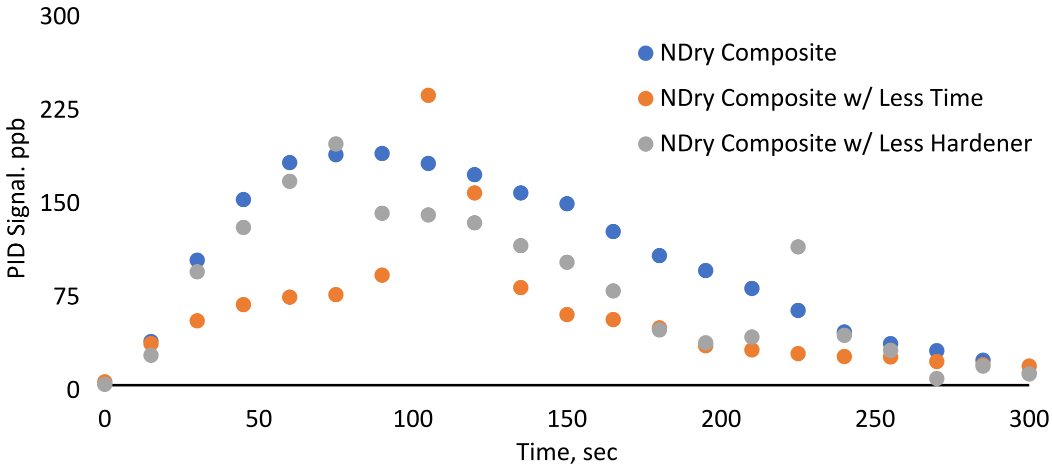

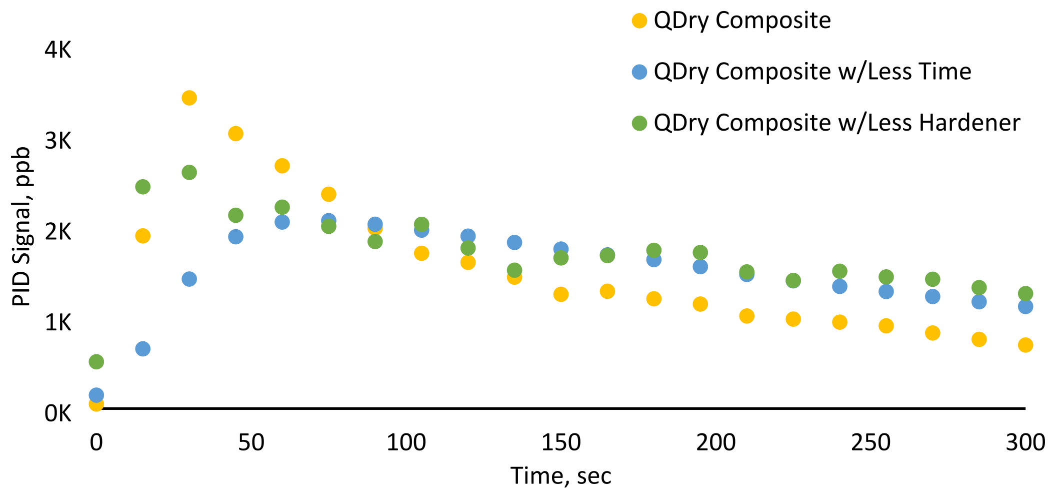

Figure SI-4. VOCs were emitted from NDry and QDry Composites into the air inside a closed container after sitting for 24 h and PID signal was measured once the container was opened

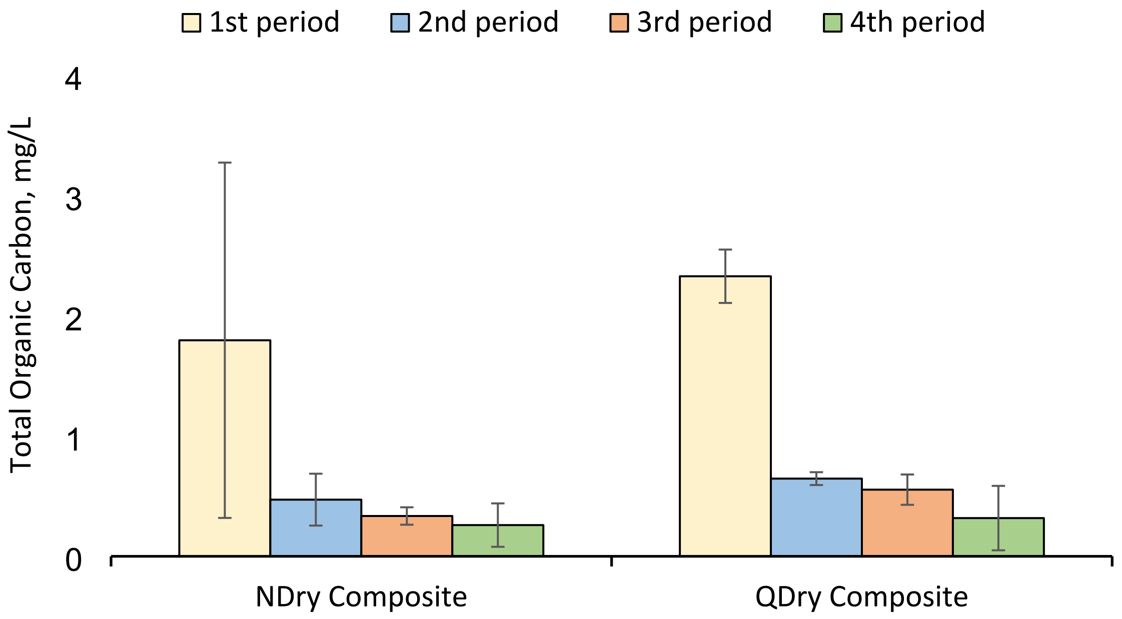

Figure SI-5. The drinking water TOC levels were examined after each exposure period for NDry and QDry composites. The first, second, and third periods were 24 h duration. The fourth period was 96 h in duration.

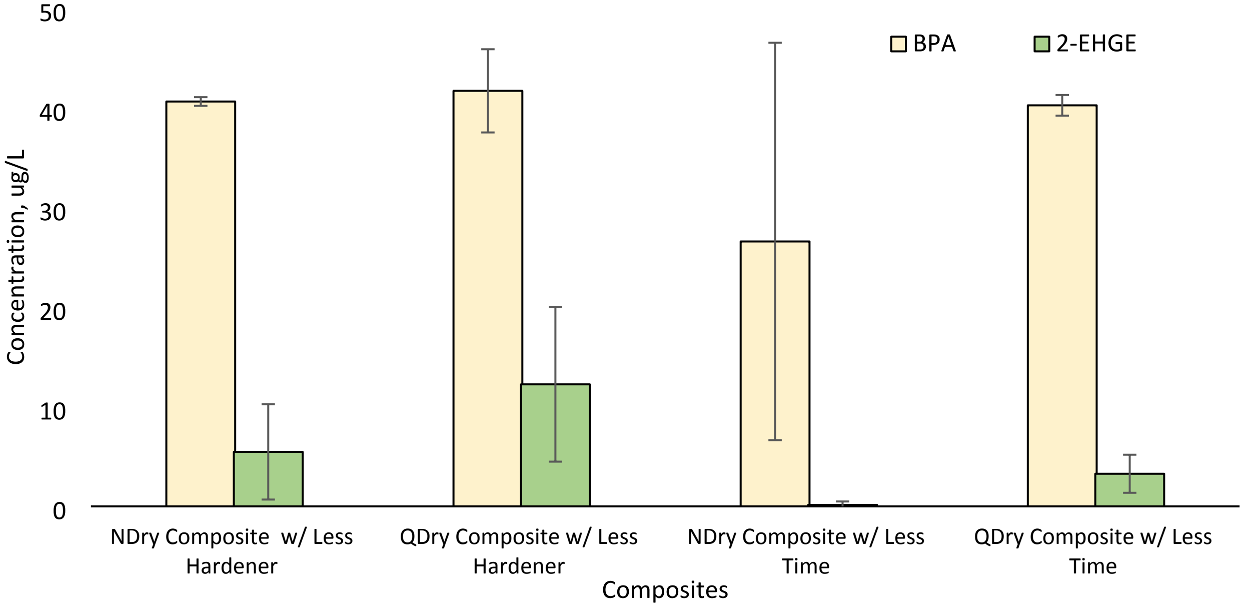

Figure SI-6. The first 24 h leachate concentration and trend for the NDry and QDry composite with less time and less hardener were like that of the NDry and QDry Composite.

Table SI-6. The presence of BPA, BADGE, and 2-EHGE in each resin, hardeners and composites in different manufacturing conditions were summarized

**Compound Info**	**Raw Material**			**Manufacturing Condition for the Composites**					
				**Normal**		**Less Time**		**Less Hardener**	
**Chemical Name**	**Resin**	**NDry-H**	**QDry-H**	**NDry-C**	**QDry-C**	**NDry-C**	**QDry-C**	**NDry-C**	**QDry-C**
2-EHGE	Yes	Yes	Yes	Yes	Yes	Yes	Yes	Yes	Yes
BPA	Yes	Yes	Yes	Yes	Yes	Yes	Yes	Yes	Yes
BADGE	Yes	No	No	No	No	No	No	Yes	Yes

2-EHGE is a viscosity reducer; BPA and BADGE are monomer related compounds; H = Hardener; C = Composite

Table SI-7. The wt% of 2-EHGE, BPA, and BADGE extractable from the composites was sometimes influenced by manufacturing conditions.

Compound Name	Mass of compounds detected in the composites					
	NDry			QDry		
	Mfg. Rec.	Less Time	Less Hardener	Mfg. Rec.	Less Time	Less Hardener
2-EHGE	3.07	3.00	3.66	6.86	9.33	5.78
BPA	0.99	0.92	0.89	1.15	1.11	1.03
BADGE	0.00	0.00	0.01	0.00	0.00	0.02

MFg. Rec. = Manufacturer recommended conditions.

Table SI-8. The wt% of organic compound residual leached into the water and compounds remaining in the composite during the study period

Compounds	Organic carbon residual leached into water, %	Organic carbon residual remaining in the composite, %
NDry Composite		
2-EHGE	0.00	100.00
BPA	0.26	99.74
BADGE	0.00	0.00
QDry Composite		
2-EHGE	0.00	100.00
BPA	0.29	99.71
BADGE	0.00	0.00

Table SI-5. Several TICs were detected in the resin, hardeners, and composites

Peak Area	Raw Materials			CIPP Composites	
	Uncured Resin	NDry Hardener	QDry Hardener	NDry Composite	QDry Composite
>100K	14	33	27	9	17
>300K	8	24	19	3	9
>500K	6	16	18	2	5
>1M	3	11	15	2	5
>5M	2	7	10	1	2
>10M	2	3	6	1	1

Table SI-6. TICS detected across materials

Peak Area	RT, min	Tentatively Identified Compound	Found In
Greater than 10,000,000	9.341	Benzyl alcohol	NDry-H, QDry-H, QDry-C
Greater than 10,000,000	10.16	Cyclohexanol, 3,3,5-trimethyl-, acetate, cis-	QDry-H
Greater than 10,000,000	13.16	[[(2-ethylhexyl) oxy] methyl]- Oxirane	Resin, NDry-C, QDry-C
Greater than 10,000,000	18.29	Heptane	Resin
Greater than 10,000,000	19.39	Butylated Hydroxytoluene	NDry-H
Greater than 10,000,000	14.41	1H-Azepin-1-amine, hexahydro-	NDry-H
Greater than 10,000,000	13.33	2,6,10,10-tetramethyl-1-Oxaspiro [4.5] dec-6-ene	QDry-H
Greater than 10,000,000	19.50	4-Octylaniline	QDry Hardener
Greater than 10,000,000	14.99	1-(3-Aminopropyl)-2-pyrrolidone	NDry-H, QDry-H
Greater than 10,000,000	19.94	2,2'-methylenebis- Phenol	NDry-H
Greater than 5,000,000	14.41	2,4-dimethyl- Heptane	NDry-H
Greater than 5,000,000	17.35	1-Piperazineethanamine	NDry-H, QDry-H
Greater than 5,000,000	13.48	Tris(2-aminoethyl) amine	NDry-H
Greater than 5,000,000	13.96	N,2-dimethyl-N-nitroso-1-Propanamine	NDry-H, QDry-H
Greater than 5,000,000	13.34	(Z)- 13-Octadecenal	NDry-H
Greater than 5,000,000	18.44	4-Methylcyclohexylamine	QDry-H
Greater than 5,000,000	18.29	4,4'-methylenebis- Cyclohexanamine	QDry-H
Greater than 5,000,000	19.89	3-(bromomethyl)- Heptane	NDry-H
Greater than 1,000,000	19.18	N-Allylaniline	QDry-H
Greater than 1,000,000	16.08	Tetraethylenepentamine	NDry-H
Greater than 1,000,000	15.96	2-ethyl-2-phenyl- Propanediamide	QDry-H
Greater than 1,000,000	19.91	Octyl thioglycolate	QDry-H
Greater than 1,000,000	16.72	1,1'-oxybis- Hexane	NDry-H
Greater than 1,000,000	18.77	2-hydroxy-, phenylmethyl ester Benzoic acid	QDry-H
Greater than 1,000,000	12.85	Salicyl alcohol	NDry-H
Greater than 1,000,000	12.88	2-amino-4-methyl- Phenol	NDry-H
Greater than 1,000,000	19.67	[[4-(1,1-dimethylethyl) phenoxy]methyl]- Oxirane	Resin, QDry-C
Greater than 1,000,000	18.81	2,4,6-tris[(dimethylamino)methyl]- Phenol	QDry-H
Greater than 500,000	9.92	o-Cresol	NDry-H
Greater than 500,000	14.07	Tetradecane	Resin, NDry-C, QDry-C
Greater than 500,000	8.51	Phenol	NDry-H, QDry-H, NDry-C
Greater than 500,000	10.23	2-methyl- Piperazine	NDry-H

	9.68	2-methyl- Phenol	NDry-H, NDry-C
	19.39	3-(2-chloro-3,3,3-trifluoro-1-propenyl)-2,2-dimethyl- ,cyano(3-phenoxyphenyl) methyl ester Cyclopropanecarboxylic acid	NDry-H
	19.39	Octane, 2,6-dimethyl-	NDry-H
	9.28	2 Ethyl Hexanol	Resin,NDry-C
Greater than 100,000	16.252	1,4-Bis(3-aminopropyl) piperazine	NDry-H
	8.13	Benzaldehyde	QDry-C
	19.06	5-heptyldihydro-2(3H)-Furanone	NDry-H
	19.06	1-Tetradecanol	NDry-H
	18.88	3-Pentanamine	QDry-C
	12.94	1-(2-methyl-1-propenyl)- Piperidine	NDry-H
	2.18	1-Butanol	QDry-H
	2.18	3,3-dimethyl- Oxetane	QDry-H
	9.21	2-Chlorocyclohexanol	NDry-C, QDry-C
	14.75	Hexadecane	Resin, QDry-C
	15.14	Heptadecane	NDry-C
	15.78	fluoroanhydride, 1-methylnonyl ester Methylphosphonic acid	NDry-H
	10.45	Hexane-2,5-diol	NDry-H
	16.36	2-Ethyl-3-methoxypyrazine	QDry -H
	15.14	Pentadecane	NDry-C, QDry-C
	16.56	1-(cyanoacetyl)- Piperidine	QDry-H
	9.28	2-ethyl-1-Hexanol	QDry-C
	10.163	3,5,5-trimethyl- Cyclohexene	QDry-H
	7.60	2-Cyclohexen-1-one	QDry-C
	13.22	2,3-Benzofurandione	QDry-H
	19.77	1-nitro-2-(octyloxy)- Benzene	NDry-H, QDry-C
	11.46	3,5-dimethyl- Phenol	NDry-H
	15.78	1-Octadecene	NDry-H
	11.46	2,4-dimethyl- Phenol / 2,5-dimethyl- Phenol	NDry-H
	14.86	1,1'-oxybis- Octane	NDry-C
	2.20	2-chloro-2-methyl- Butane	NDry-H
	9.36	N-[(phenylmethoxy)carbonyl]- dl-Leucine	NDry-C

	18.41	1-bromo- Tetradecane	QDry-C
	12.97	[2R-[2.alpha., 6.alpha.(E)]- 2-Methyl-6-(1-p ropenyl) piperidine	QDry-H
	12.88	Tridecane	Resin
	14.75	2,6,10-trimethyl- Dodecane	Resin
	13.62	Hexacosane	Resin
	18.46	2-bromo- Octane	Resin, NDry-C, QDry-C
	13.34	cis-9-Hexadecenal	NDry-H
	9.36	Benzenemethanol	NDry-C
	19.75	Isobornyl propionate	Resin
	12.80	1,5-Hexadien-3-ol	Resin
	17.80	p-Cresol	NDry-H
	9.93	2-methyl- Phenol	QDry-H
	17.97	4,4'-methylenebis [2,6-dimethyl- Phenol	QDry-H

RT = retention time

SI-References

Alben, K., Bruchet, A., & Shpirt, E. (1989). Leachate from Organic Coating Materials Used in Potable Water Distribution Systems. The Water Research Foundation. Denver, Colorado, USA. https://www.waterrf.org/research/projects/leachate-organic-coating-materials-used-potable-water-distribution-systems

Bae, B., Jeong, J. H., & Lee, S. J. (2002). The Quantification and Characterization of Endocrine Disruptor Bisphenol-A Leaching from Epoxy Resin. *Water Science & Technology*, 46(11-12), 381-387. https://doi.org/10.2166/wst.2002.0766

Bruchet, A., Elyasmino, N., Decottignies, V., & Noyon, N. (2014). Leaching of Bisphenol A and F from New and Old Epoxy Coatings: Laboratory and Field Studies. *Water Supply*, 14(3), 383-389. https://doi.org/10.2166/ws.2013.209

Deb, A., McCammon, S., Snyder, A. M., & Dietrich, A. M. (2010). Impact of Lining Materials on Water Quality. The Water Research Foundation. Denver, Colorado, USA. https://www.waterrf.org/research/projects/impacts-lining-materials-water-quality-0

Heim, T. H., & Dietrich, A. M. (2007). Sensory Aspects of Drinking Water in Contact with Epoxy Lined Copper Pipe. *Water Science & Technology*, 55(5), 161-168. https://doi.org/10.2166/wst.2007.175

Jackson, P. J., Warren, I. C., & James, H. A. (2007). The Long-Term Migration of Substances from In-Situ Applied Epoxy Resin Coatings: Final Report to the Drinking Water Inspectorate, DWI 7369/1. WRc-NSF Ltd., Reading, UK.

Kosaka, K., Asami, M., & Yamada, T. (2002). Elution of Bisphenol A from Epoxy Resin-Coated Pipes Used in Water Supply Process. *Water Science & Technology*, 46(11-12), 381-387. https://doi.org/10.2166/wst.2002.0766

Kosaka, K., Hayashida, T., Terasaki, M., Asami, M., Yamada, T., Itoh, M., & Akiba, M. (2012). Elution of Bisphenol A and Its Chlorination By-Products from Lined Pipes in Water Supply Process. *Water Supply*, 12(6), 791-798. https://doi.org/10.2166/ws.2012.055

NSF International. (2016). NSF/ANSI 61-2016: Drinking Water System Components - Health Effects. Ann Arbor, Michigan, USA.

Rajasärkkä, J., Pernica, M., Kuta, J., Lašňák, J., Šimek, Z., & Bláha, L. (2016). Drinking Water Contaminants from Epoxy Resin-Coated Pipes: A Field Study. *Water Research*, 103, 133-140. https://doi.org/10.1016/j.watres.2016.07.027

Randtke, S. J., Adams, C. D., & Carter, R. E. (2017). Evaluation of Lead Service Line Lining and Coating Technologies. The Water Research Foundation. Denver, Colorado, USA.

Rigal, S., & Danjou, J. (1999). Tastes and Odors in Drinking Water Distribution Systems Related to the Use of Synthetic Materials. *Water Science & Technology*, 40(6), 203-208. https://doi.org/10.2166/wst.1999.0299

Romero, J., Ventura, F., & Gomez, M. (2002). Characterization of Paint Samples Used in Drinking Water Reservoirs: Identification of Endocrine Disruptor Compounds. *Journal of Chromatographic Science*, 40(4), 191-197. https://doi.org/10.1093/chromsci/40.4.191
